# New marking method involving a light-emitting diode and power source device to localize gastrointestinal cancer in laparoscopic surgery

**DOI:** 10.1038/s41598-019-41981-w

**Published:** 2019-04-02

**Authors:** Yuma Wada, Norikatsu Miyoshi, Shiki Fujino, Masayuki Ohue, Masayoshi Yasui, Yusuke Takahashi, Hidekazu Takahashi, Junichi Nishimura, Yuya Takenaka, Kazuhiro Saso, Akira Tomokuni, Keijiro Sugimura, Hirofumi Akita, Hidenori Takahashi, Shogo Kobayashi, Takeshi Omori, Hiroshi Miyata, Masahiko Yano

**Affiliations:** 1 Department of Surgery, Osaka International Cancer Institute, 3-1-69, Ohtemae, Chuo-ku, Osaka, 541-8567 Japan; 2 Department of Gastroenterological Surgery, Graduate School of Medicine, Osaka University, 2-2-E2 Yamadaoka, Suita, Osaka, 565-0871 Japan

## Abstract

Although the preoperative endoscopic marking method using dye is widely used, the dye can spread into the tissue or abdominal cavity, inducing the inflammation and leading to the wrong dissection. We developed a novel marking method using an endoscopic clip with a light emitting diode (LED) and a power source device to detect the accurate location of the site of interest. We performed this new marking method in three patients with gastrointestinal cancers. We placed an endoscopic clip with an LED on the gastrointestinal mucosa and used a power source device outside of the human body to detect the LED. We detected the clip with the LED using the power source device. We also confirmed the usefulness of this clip in three of three (100%) patients with colorectal and gastric cancer. We developed a novel marking device using an LED to identify an objective location successfully.

## Introduction

Laparoscopic surgery of the digestive organs has been widely performed for more than 25 years^1^. Laparoscopic techniques have been effectively applied for gastrointestinal surgery in many institutions, resulting in reduced blood loss, a shorter hospitalization period, decreased postoperative pain, faster postoperative recovery, and improved quality of life compared with general open surgery^2–4^. However, accurate localization of tumor lesions in patients with early cancer is difficult because direct contact with the organ is not possible. Therefore, marking the tumor lesion during preoperative endoscopy and determining the accurate oncologic resection range are necessary^5^. The usual preoperative marking method is currently a tattooing method in which India ink is injected into the submucosal membrane layer^6^. However, this method has several problems such as inflammation, perforation, and spreading of the ink^7,8^. We previously reported the surgical usefulness of indocyanine green as a safer alternative^9^. However, the problem of dye diffusion has not been resolved.

Endoscopic clips are palpable during open surgery. However, these clips are invisible from the outside of the serosal walls of the gastrointestinal tract and are not palpable during laparoscopic surgery. Although the best method of identifying tumor lesions is intraoperative endoscopy, it is not recommended in terms of cost, the need for additional human power, and extension of the operation time. To identify the location of the tumor and determine the most accurate resection range, we previously reported a marking method that involves the use of an integrated circuit tag^10^. We subsequently improved this marking method and developed a new marking method. This new method involves an endoscopic clip combined with a light-emitting diode (LED) and power source using electromagnetic power transfer to identify a precise location during laparoscopic surgery. In the present study, we assessed the usefulness and safety of this marking method in human gastrointestinal tissue during surgery.

## Materials and Methods

### LED marker

**Figure 1:**
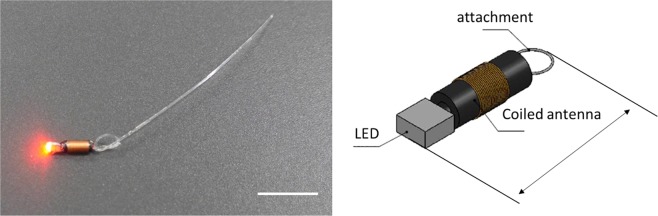
Design of the light-emitting diode (LED) marker. The LED is attached to the tip of a coiled antenna and coated with paraxylene rubber. Scale bar: 10 mm.

**Figure 2:**
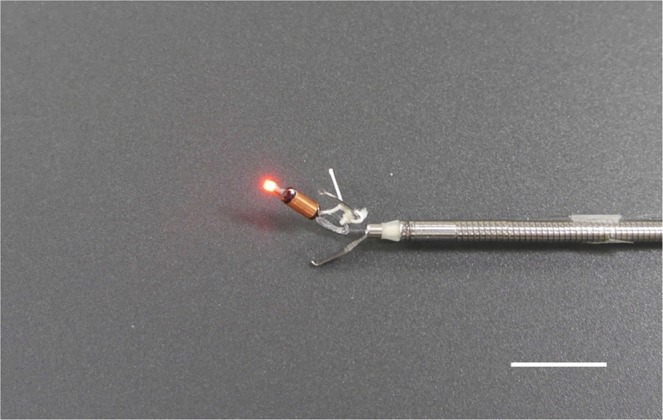
Photograph of the light-emitting diode (LED) marker with a string. The LED marker is linked to an endoscopic clip with a string. Scale bar: 10 mm.

We developed an LED marker that is attached to the tip of a coiled antenna and coated with paraxylene rubber (Fig. 1). The LED marker is linked to an endoscopic clip with a string (Fig. 2). An endoscopic clip (model HX-610-090; Olympus Medical Systems Corporation, Tokyo, Japan) is used to attach and maintain the endoscopic clip with the LED marker on the mucosal epithelium at the objective location. The size of the coiled antenna with the LED is 9 × 2 × 2 mm, which allows it to pass through the forceps aperture of a gastrointestinal endoscope.

### Power source device

**Figure 3:**
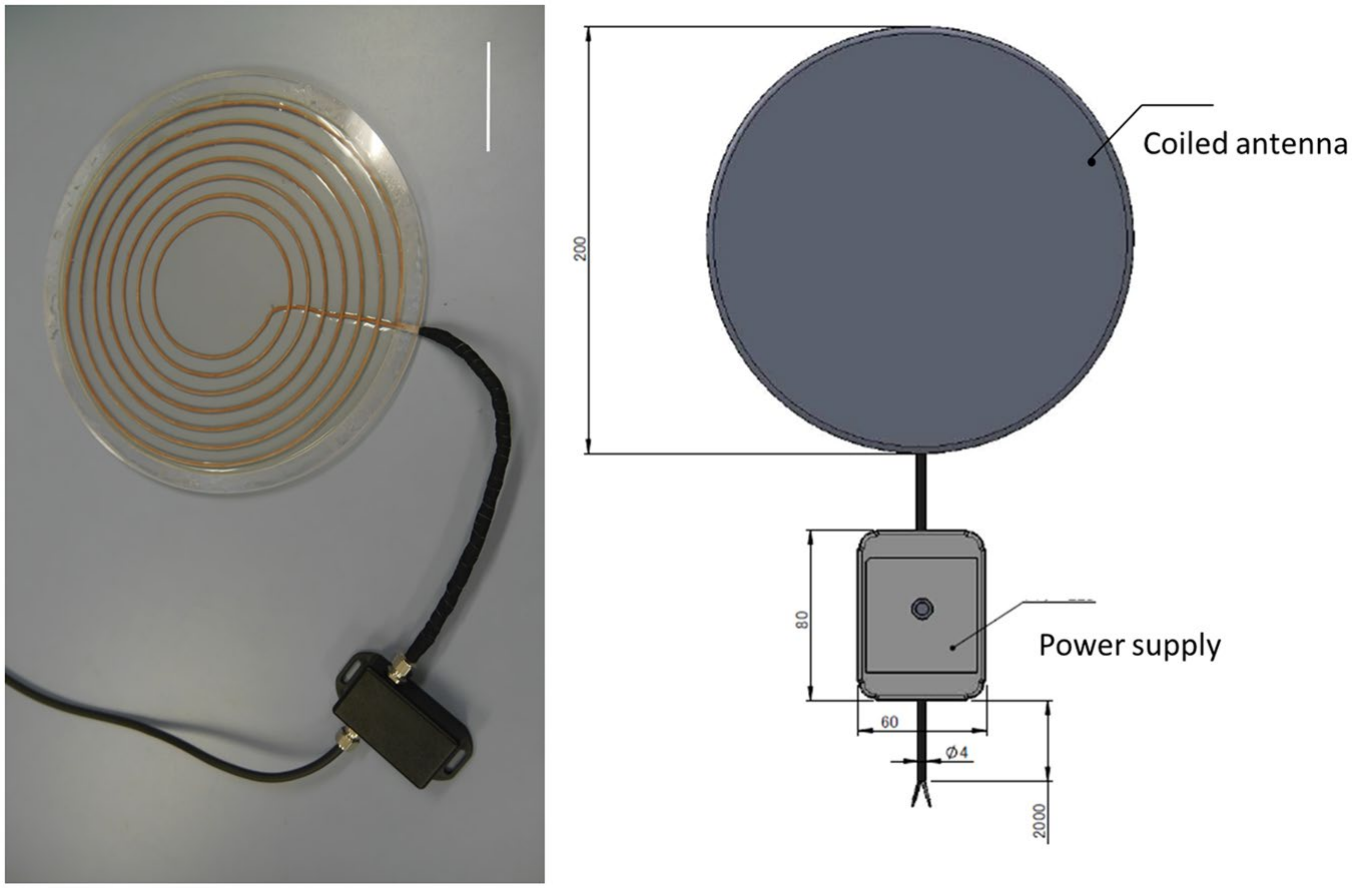
Design of the power source device. The power source device is an induction coil antenna. The light-emitting diode, which is connected to the coiled antenna, can be sensed with light in an electromagnetic field and reacts to an electric wave that is emitted from the induction coil antenna. Scale bar: 50 mm.

The power source device is an induction coil antenna. An LED connected to a coiled antenna can be sensed with light in an electromagnetic field that reacts to an electric wave emitted from an induction coil antenna (Fig. 3). The power source device is covered with a sterilized bag and connected to a power supply. The power source device is designed to detect an electric wave outside of the human body during laparoscopic surgery. The LED marker can be detected through the intestinal wall and create light upon detection of an object. The LED lights up only when the power source antenna detects it. These devices were made in compliance with the Japanese Radio Act and the Japanese Ministry of Internal Affairs and Communications. The LED marker is a noninvasive and useful detectable marker for identifying an objective location.

### Clinical examination

**Figure 4:**
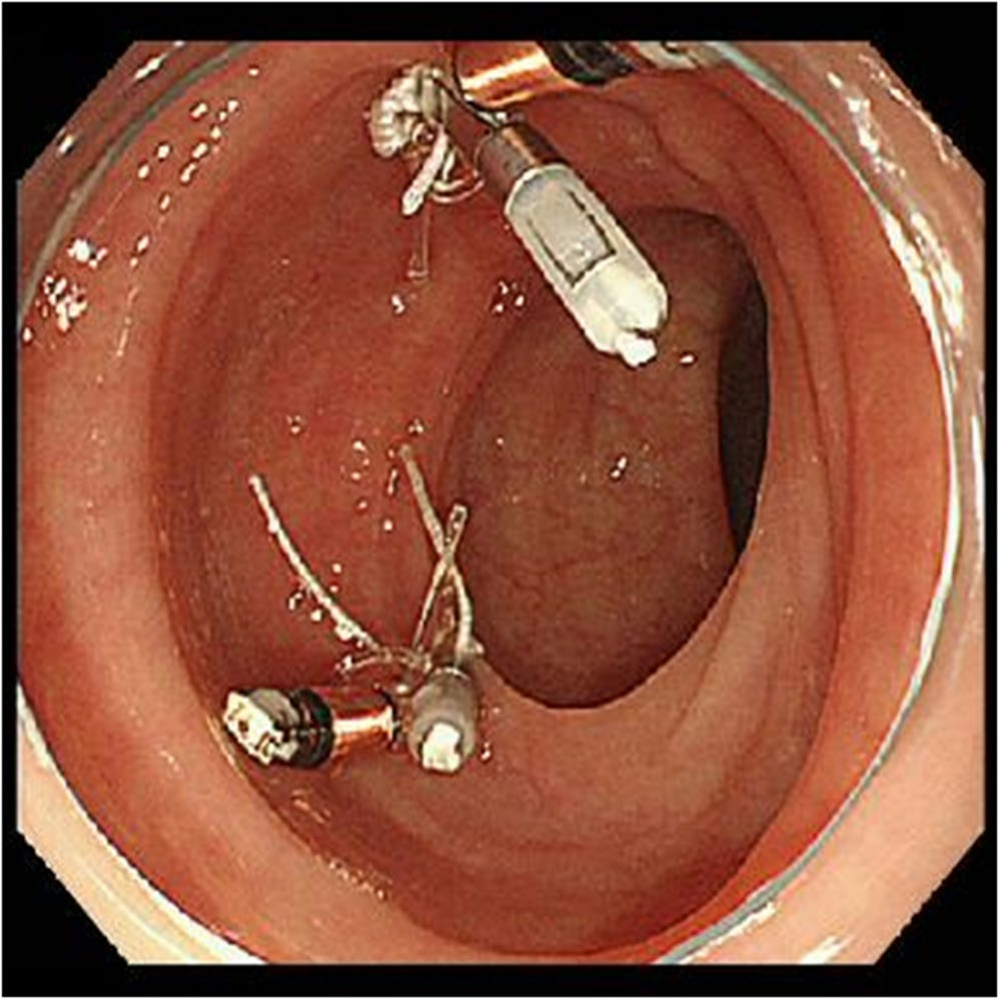
Preoperative clinical examination. We placed an endoscopic clip with a light-emitting diode on the colonic mucosa of a patient during a preoperative endoscopic examination.

Two LED markers were placed on the gastrointestinal mucosa to determine their detectability (Fig. 4). These two markers were placed opposite to each other (180 degrees) to avoid the mesenteric and anal sides of the lesion. We assessed the detectability of the LED markers in two patients with colorectal cancer and in one patient with gastric cancer by two other laparoscopic surgeons (Table 1). Two sets of an endoscopic clip with an LED were placed on the gastrointestinal mucosa from 3 days to 1 day before the operation. Laparoscopic surgery was performed, and we evaluated the LED marker using the power source device outside of the patient’s body. This study was approved by the Institutional Review Board of the Osaka International Cancer Institute (No. 1512046211; UMIN000032204; Date of registration, February 2, 2016). Written informed consent was obtained from all patients, and all experiments were performed in accordance with relevant guidelines and regulations.

**Table 1:** List of patients and the locations of gastrointestinal cancers examined by the LED marking method.

Patient number	Age (yrs)/Gender	BMI (kg/m^2^)	Tumor location	Detectable or not
1	68/Female	20.2	Sigmoid colon	Detectable
2	64/Male	26.2	Sigmoid colon	Detectable
3	60/Male	18.4	Antrum (Stomach)	Detectable

BMI: Body Mass Index.

## Results

**Figure 5:**
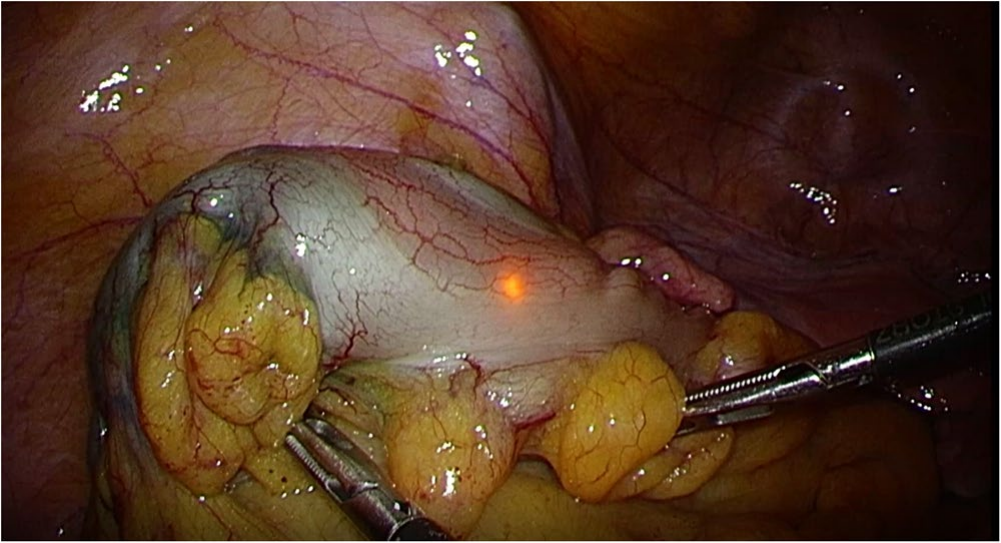
Clinical examination during laparoscopic surgery. We used a power source device outside of the patient’s body to detect the light-emitting diode (LED). The LED marker was successfully detected and its precise position was indicated by light.

The LED markers were detected using the power source device during the operation in all three of the patients’ gastrointestinal organs. The LED marker was powered outside of the body. We validated the LED marker, which was able to be identified from the serosal side of the gastrointestinal organs (Fig. 5). Detection of the LED marker was accurate. In the resected specimen, the endoscopic clip was located on the mucosa and indicated an accurate resection range. Histologic evaluation of the surgical specimens showed no complications such as inflammation, necrosis, or fibrotic reaction. Using the power source device, the LED marker was successfully detected and its precise position was indicated by light (Movie 1). Accurate laparoscopic resection of the lesion was able to be performed, and the clips aided in accurate laparoscopic resection.

## Discussion

Accurate localization of tumors during laparoscopic surgery is important. Detecting the precise location of a tumor shortens the operation time and reduces operative invasion, leading to faster postoperative recovery and establishment of an oncological safety margin^5^.

Preoperative marking is currently used for localization in laparoscopic surgery, and marking techniques include preoperative endoscopic clipping, endoscopic tattooing, and intraoperative endoscopy^11,12^. Among these three methods, the most common marking method is endoscopic tattooing. India ink or indocyanine green is injected into the submucosal layer of the gastrointestinal wall^9,13–15^. With the tattooing method, however, the dye spreads into the submucosal layer or abdominal cavity, making identification of a precise location more difficult^16,17^. Moreover, some studies have shown that leakage of India ink into the abdominal cavity induces severe complications, such as peritonitis, and the reported complication rate was 0.22%^18–20^. Intraoperative endoscopy is a useful approach, but it requires an additional endoscope, an endoscopist, and a longer operation time. Laparoscopic surgery is difficult because it sometimes causes air inflation of the intestine^21,22^. A preoperative marking clip is the easiest and most noninvasive method among all preoperative marking methods. A recent study showed that fluorescence-coated clips (indocyanine green and CF™790; Biotium, Hayward, CA, USA) were visible with near-infrared fluorescence imaging in a porcine model of laparoscopic surgery^23,24^. However, this method requires an atypical near-infrared fluorescence imaging system to detect the fluorescence-coated clips and approval for clinical use.

We previously reported a marking method that involves an integrated circuit tag^10^. Furthermore, we improved our previous device to create a novel marking method with an LED and power source using electromagnetic power transfer to identify a precise location during laparoscopic surgery. In our previous study, we evaluated the efficacy and safety of the present technology in a porcine model of laparoscopic surgery^10^. The detectable distance between the LED marker and power source was about 20 cm. This suggests that our device can be detected even in patients with a higher body mass index to move the marked colon or the power source antenna outside of the body. The LED activates only when the power source antenna detects it, preventing the LED light from generating any heat.

This is the first report of a new marking clip with an LED that can accurately identify an objective location and is noninvasive for intraoperative use in humans. However, our study has some limitations. Commercializing the product and limiting costs to allow its general clinical application are necessary. In the present study, we placed the LED marker from 3 days to 1 day before the operation; for longer durations of placement, the timeline between colonoscopic placement of the marker and surgery should be evaluated. Our novel device was validated in only a few patients with gastrointestinal cancer. However, this tool can help physicians to precisely detect target locations in laparoscopic surgery, leading to better outcomes for patients. Clinical trials will be conducted to evaluate the usefulness, examining the physicians’ stress and surgical outcomes for the future.

## Conclusions

We developed a novel marking device using an endoscopic clip with an LED and power source device to precisely detect an objective location. We successfully demonstrated the usefulness of this clip with an LED and antenna device for detection of gastrointestinal cancer in patients.

## Supplementary information

Movie 1

## References

[1] Kano N (1994). Laparoscopic cholecystectomy: a report of 409 consecutive cases and its future outlook. Surg Today..

[2] Yamamoto S (2014). Short-term surgical outcomes from a randomized controlled trial to evaluate laparoscopic and open D3 dissection for stage II/III colon cancer: Japan Clinical Oncology Group Study JCOG 0404. Ann Surg..

[3] Chan MK (2008). Erratum: Systematic review on the short term outcome of laparoscopic resection for colon and rectosigmoid cancer. Colorectal Dis..

[4] Tjandra JJ, Chan MK (2006). Systematic review on the short-term outcome of laparoscopic resection for colon and rectosigmoid cancer. Colorectal Dis..

[5] Montorsi. M (1999). Original technique for small colorectal tumor localization during laparoscopic surgery. Dis Colon Rectum..

[6] Ponsky JL, King JF (1975). Endoscopic marking of colonic lesions. Gastrointest Endosc..

[7] Dell’Abate P (1999). Endoscopic preoperative colonic tattooing: a clinical and surgical complication. Endoscopy..

[8] Del Rio P, Dell’Abate P (2003). Complication of an endoscopic tattoo. Endoscopy..

[9] Miyoshi N (2009). Surgical usefulness of indocyanine green as an alternative to India ink for endoscopic marking. Surg Endosc..

[10] Wada Y (2016). Endoscopic marking clip with an IC tag and receiving antenna to detect localization during laparoscopic surgery. Surg Endosc..

[11] Cho YB (2007). Tumor localization for laparoscopic colorectal surgery. World J Surg..

[12] Beretvas RI, Ponsky J (2001). Endoscopic marking: an adjunct to laparoscopic gastrointestinal surgery. Surg Endosc..

[13] Luigiano C (2012). Endoscopic tattooing of gastrointestinal and pancreatic lesions. Adv Ther..

[14] Fu KI (2001). A new endoscopic tattooing technique for identifying the location of colonic lesions during laparoscopic surgery: a comparison with the conventional technique. Endoscopy..

[15] Trakarnsanga A, Akaraviputh T (2011). Endoscopic tattooing of colorectal lesions: Is it a risk-free procedure?. World J Gastrointest Endosc..

[16] Askin MP, Waye JD, Fiedler L, Harpaz N (2002). Tattoo of colonic neoplasms in 113 patients with a new sterile carbon compound. Gastrointest Endosc..

[17] Sawaki A (2003). A two-step method for marking polypectomy sites in the colon and rectum. Gastrointest Endosc..

[18] Park JW (2008). The usefulness of preoperative colonoscopic tattooing using a saline test injection method with prepackaged sterile India ink for localization in laparoscopic colorectal surgery. Surg Endosc..

[19] Price N (2000). Safety and efficacy of India ink and indocyanine green as colonic tattooing agents. Gastrointest Endosc..

[20] Nizam R, Siddigi N, Landas SK, Kaplan DS, Holzapple PG (1996). Colon tatooing with India ink: benefits, risks, and alternatives. Am J Gastroenterol..

[21] Hyung WJ (2015). Intraoperative tumor localization using laparoscopic ultrasonography in laparoscopic-assisted gastrectomy. Surg Endosc..

[22] Park DJ (2005). Intraoperative gastroscopy for gastric surgery. Surg Endosc..

[23] Takeyama H (2014). A novel endoscopic fluorescent clip visible with near-infrared imaging during laparoscopic surgery in a porcine model. Surg Endosc..

[24] Choi Y (2011). A novel endoscopic fluorescent clip for the localization of gastrointestinal tumors. Surg Endosc..

